# Implementation of cross-sector partnerships: a description of implementation factors related to addressing social determinants to reduce racial disparities in adverse birth outcomes

**DOI:** 10.3389/fpubh.2023.1106740

**Published:** 2023-06-16

**Authors:** Bridgette E. Blebu, Patrick Y. Liu, Maura Harrington, William Nicholas, Ashaki Jackson, Erin Saleeby

**Affiliations:** ^1^The Lundquist Institute for Biomedical Innovation at Harbor UCLA Medical Center, Torrance, CA, United States; ^2^Center for Healthier Children, Families, and Communities, University of California, Los Angeles, Los Angeles, CA, United States; ^3^Department of Pediatrics, David Geffen School of Medicine, University of California, Los Angeles, Los Angeles, CA, United States; ^4^Center for Nonprofit Management, Los Angeles, CA, United States; ^5^Center for Health Impact Evaluation, Los Angeles Country Department of Public Health, Los Angeles, CA, United States; ^6^Women’s Health Programs and Innovations, Los Angeles County Department of Health Services, Alhambra, CA, United States

**Keywords:** implementation, health disparities, prenatal care, social detereminants, cross-sector partnership

## Abstract

**Introduction:**

Traditional perinatal care alone cannot address the social and structural determinants that drive disparities in adverse birth outcomes. Despite the wide acceptance of partnerships between healthcare systems and social service agencies to address this challenge, there needs to be more research on the implementation factors that facilitate (or hinder) cross-sector partnerships, particularly from the perspective of community-based organizations. This study aimed to integrate the views of healthcare staff and community-based partner organizations to describe the implementation of a cross-sector partnership designed to address social and structural determinants in pregnancy.

**Methods:**

We used a mixed methods design (in-depth interviews and social network analysis) to integrate the perspectives of healthcare clinicians and staff with those of community-based partner organizations to identify implementation factors related to cross-sector partnerships.

**Results:**

We identified seven implementation factors related to three overarching themes: relationship-centered care, barriers and facilitators of cross-sector partnerships, and strengths of a network approach to cross-sector collaboration. Findings emphasized establishing relationships between healthcare staff, patients, and community-based partner organizations.

**Conclusion:**

This study provides practical insights for healthcare organizations, policymakers, and community organizations that aim to improve access to social services among historically marginalized perinatal populations.

## 1. Introduction

Perinatal care alone is insufficient to address the unmet social needs, such as food insecurity and housing instability that contribute to disparities in adverse birth outcomes (e.g., preterm birth, infant mortality) among vulnerable populations. Because social determinants of health (SDOH) continue to drive health disparities, collaborations between healthcare and social service sectors are becoming increasingly common in healthcare organizations to mitigate health-related social stressors and achieve equity (1–3). These cross-sector partnerships aim to bridge fragmented care systems and contend with unmet social needs by forging partnerships between often siloed sectors (4). For instance, medical-financial partnerships that establish collaborations between healthcare and financial service organizations to address financial stressors more broadly (and not limited to healthcare expenses) can reduce financial stress, particularly during critical periods such as pregnancy (5). Other studies demonstrate that connecting older adults to social services in health systems may reduce hospitalizations and emergency room visits (6).

As the adoption of cross-sector partnerships continues to increase, many studies have documented their core components (7–13). For example, Liu et al. (9) describe specific foundational structures, such as linked data and communication platforms, and a shared theory of change (i.e., a clear pathway through which a cross-sector partnership will improve outcomes) that can support the functionality of cross-sector partnerships. Some studies describe the importance of policies to support collaboration and the quality of relationships within a partnership and financial investments to incentivize and facilitate partnerships (12, 13). Others note the importance of buy-in and demonstrated commitment from a broad network of agencies (10). However, cross-sector partnerships remain challenging to implement despite identifying these core components.

Structural challenges related to organizational culture, funding, and different approaches to service delivery make cross-sector partnership implementation complex and threaten sustainability (14). Some studies have shown that the extent healthcare organizations engage in cross-sector partnerships can vary depending on hospital characteristics. For instance, Noh et al. (15) found that among hospitals in United States counties with the highest socioeconomic disadvantage, larger hospitals, teaching hospitals, and hospitals in health systems were significantly more likely to partner with non-health sector organizations to address unmet social needs. Similarly, Nelson (16) found that health department participation in cross-sector partnerships was more likely if resource sharing (e.g., shared personnel) was already taking place and written agreements were established. These findings have important implications for equitable access to cross-sector partnerships among patients.

Understanding the perspectives and experiences of community-based organizations and other partner agencies can also unpack factors associated with the implementation and sustainability of cross-sector partnerships. Agonafer et al. (17) found that while community-based organizations valued partnerships with health systems, they desired more equitable collaborations that included bi-directional exchanges of information and shared-decision making related to the design and implementation of the partnerships. Other studies have documented perceived challenges related to cross-sector partnerships among community-based organizations associated with losing autonomy and distinct approaches to care through partnerships with healthcare systems (18, 19). These findings speak to the relational and technical challenges of building and sustaining equitable cross-sector partnerships, potentially limiting their impact on upstream drivers and health outcomes.

Additional research is needed to describe cross-sector partnership implementation and to shed light on implementation strategies that support cross-sector partnerships, particularly in serving perinatal populations with unmet social needs. This study aims to examine the implementation of an enhanced prenatal care program called MAMA’S Neighborhood that incorporates cross-sector partnerships to address social determinants associated with adverse birth outcomes among Medicaid-eligible perinatal clients. To achieve this objective, we describe relevant implementation factors (i.e., processes, barriers, and facilitators) related to cross-sector partnerships. We use a mixed methods (in-depth interviews and social network analysis) design (20, 21) to integrate the perspectives of healthcare clinicians and staff with those of community-based partner organizations to provide a comprehensive exploration of cross-sector partnership implementation from the perspective of key implementors.

## 2. Methods

### 2.1. Study setting

Medicaid covers over 95% of births in the Los Angeles County Department of Health Services, one of the largest safety net systems in the United States. Preterm birth rates are twice as high among Los Angeles County Department of Health Services clients (18.5%) compared to births to women with Medicaid coverage in Los Angeles County (9%) (22, 23). Similar inequities between these two populations exist with social stressors such as housing instability, intimate partner violence, and food insecurity. Comprehensive interventions to address social, medical, and behavioral determinants known to impact birth outcomes in this health system are critical.

Within the Los Angeles County Department of Health Services, Maternity Assessment Management Access and Service synergy throughout the Neighborhood (MAMA’S Neighborhood) is an innovative, multi-sector initiative that seeks to address social determinants of health known to contribute to the risk of adverse birth outcomes. The program includes linkage to services, including housing, food assistance, health education, mental health treatment, and substance use disorder treatment. To address the siloed service landscape in Los Angeles County, MAMA’S Neighborhood also aims to foster cross-sector collaboration and strengthen the integration of health and social service systems by bringing together three key service sectors: public health, health care, and social services, beyond the clinic and in community settings. The MAMA’S Neighborhood approach includes team-based care that integrates traditional perinatal care providers with a MAMA’S Neighborhood Care team (hereafter MAMA’S Neighborhood staff), which includes social workers, community health workers (called Care Coordinators), mental health providers, and health educators. MAMA’S Neighborhood staff support care coordination and continuity with a global risk screening for social stressors and individual care planning (Care Coordinators), mental and behavioral health (social workers, mental health providers), and pregnancy/nutrition education (health educators). Lastly, MAMA’S Neighborhood also includes a network of partner organizations (public health, social services, and health care agencies) that support the referral process to address unmet social needs. Currently, MAMA’S Neighborhood is the standard of care for all perinatal clients. Early evidence on MAMA’S Neighborhood impact suggests significant pre/post reductions in preterm birth rates following the implementation of cross-sector partnerships and collaborative care (14.9 vs. 15.7%), particularly for Black women (18.2 vs. 9.1%) (24).

### 2.2. Data

Data sources for this study include in-depth interviews with MAMA’S Neighborhood clinicians and staff (*N* = 18) and social network survey data collected from MAMA’S Neighborhood partner organizations (*N* = 19). All data were collected between May 2019 and May 2021. This study received human subject’s research approval from the Los Angeles County Department of Public Health institutional review board (IRB # 2013-08-451).

#### 2.2.1. In-depth interviews

A local non-profit research and evaluation organization conducted in-depth interviews with MAMA’S Neighborhood staff. The overall goal of the interviews was to understand the context of MAMA’S Neighborhood program implementation among staff. Guiding questions for the interviews included the following:

- What does the intake process entail? To what extent does it serve its intended purpose?
- Where does the collaboration among MAMA’S Neighborhood staff occur? How does collaboration contribute to engaging the patient and the overall success of the patient’s health, pregnancy, delivery, and motherhood experience, if at all?
- What contributes to a successful referral to resources and programs among the MAMA’S Neighborhood partner organizations?
- What strategies effectively maintain a patient’s engagement with the MAMA’S Neighborhood?

The interviews were conducted virtually and audio recorded. Transcripts were developed verbatim and deidentified before analysis.

#### 2.2.2. MAMA’S neighborhood network analysis

We collected network data among MAMA’S Neighborhood partner organizations using the Visual Network Labs PARTNER Community Partner Relationship Management Software (CPRM) platform (25). The PARTNER CPRM platform is an online tool that uses social network analysis to facilitate the collection, analysis, and interpretation of collaboration data in community-based networks. PARTNER generates data to identify partners, quantify relationships, and compute social network metrics related to the quality of relationships (e.g., trust and value) within a network. The MAMA’S Neighborhood network analysis included survey questions related to perceptions of MAMA’S Neighborhood success with facilitating referrals through cross-sector partnership and overall experiences collaborating within the MAMA’S Neighborhood partner organizations network. Participants were invited via email to participate in the network surveys. Each participant received follow-up emails to increase the response rates.

### 2.3. Analysis

We aimed to triangulate the perspectives of MAMA’S Neighborhood clinicians and staff (in-depth interview data) and MAMA’S Neighborhood partner organizations (network analysis data) to better understand the implementation of cross-sector partnerships. We coded data thematically using inductive codes that emerged from the interviews and the network data to generate themes about factors related to cross-sector partnership implementation (e.g., referral processes logistics, communication among partner agencies, and information sharing between care coordinators and partner agencies). All coding was conducted using Atlas.ti 9.

After coding each data source, we followed triangulation methodology of Farmer et al. (26) to develop a triangulation protocol that would guide the synthesis of codes from each source. The triangulation protocol consisted of a five-step process. We first sorted the codes identified in each data source into similar themes around implementing cross-sector partnerships (Step 1: sorting). Next, we coded deductively to evaluate the level of convergence between both data sources within each of the themes, using the following coding scheme: “full agreement,” “partial agreement,” “disagreement,” and “silence.” (Step 2: convergence coding).

We evaluated convergence based on (1) descriptions of implementation factors (e.g., factors that hindered implementation, factors that improved implementation) and (2) mechanistic descriptions of how each factor shaped cross-sector partnership implementation. We used the “full agreement” code for implementation factors with complete convergence on substantive and mechanistic descriptions and “partial agreement” for convergence of either the substantive or mechanistic description. For instance, if MAMA’S Neighborhood staff and partner organizations described heavy client caseloads as a barrier to implementing cross-sector partnerships and specifically described heavy caseloads as hindering their ability to engage more than one partner organization in the network, we would code this factor as “full agreement.” Alternatively, if MAMA’S Neighborhood staff reported that heavy client caseloads hindered their ability to address all clients’ social needs while partner agencies shared that they could not engage with more than one agency due to heavy caseloads, we would code this as “partial agreement.” We used the “disagreement” code for instances of divergence when neither substantive nor mechanistic descriptions converge. Lastly, we used the “silence” code when we identified a factor in one data set but not the other. Two authors then reviewed the convergence findings, clarified interpretations, and finalized the coding (Steps 3–5).

## 3. Results

**Figure 1:**
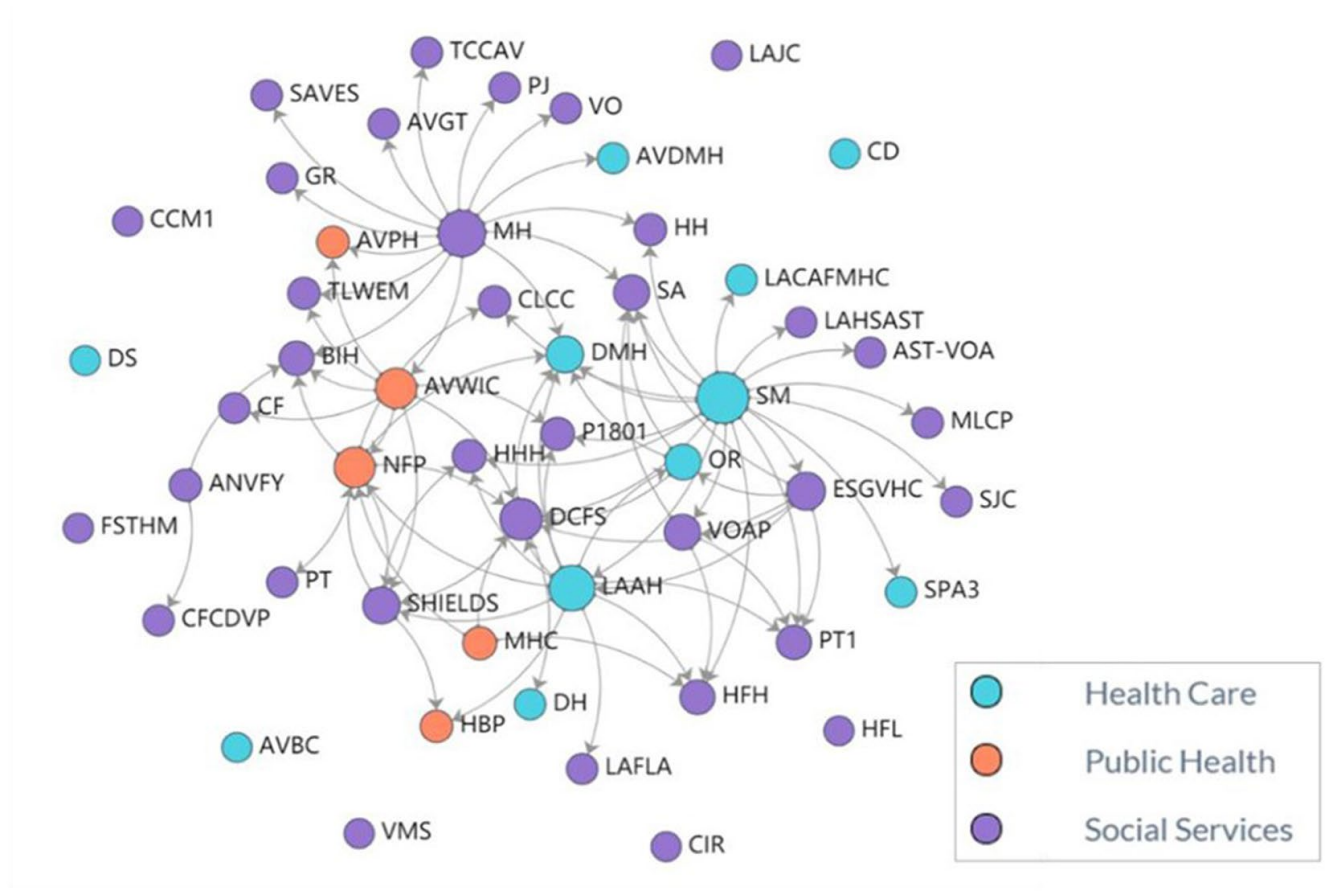
MAMA’S neighborhood partnerships (*N* = 80 unique partnerships). MAMA’S staff identified 51 organizations (nodes) as part of the network. These organizations identified 80 unique partnerships (arrows). Larger nodes indicate organizations with a greater number of relationships. The network includes 11 (22%) healthcare organizations, 35 (68%) social services organizations, and 5 (10%) public health organizations throughout Los Angeles County. A list MAMA’S Neighborhood partner organization names is included in Appendix 1.

Overall, we identified seven implementation factors to address social determinants of health in perinatal care through cross-sector partnerships. These factors were related to three broader themes: relationship-based care, cross-sector partnership implementation barriers and facilitators, and strengths of a network approach to cross-sector partnership. Most implementation factors were in full agreement, except for the three factors that were silent (*n* = 2) or in partial agreement (*n* = 1). Beyond the implementation factors, we identified broader themes that showed high fidelity to the MAMA’s Neighborhood approach to team-based perinatal care for addressing unmet social needs. The network analysis yielded a network of 51 organizations (nodes) identified by MAMA’S Neighborhood staff, which corresponded to 80 unique relationships (arrows) among the partner organizations (Figure 1). Larger nodes depict organizations with a more significant number of relationships. Of the 51 organizations, 40 were invited to participate in the survey (11 organizations did not have updated contact information), and 19 responded (48% response rate, Table 1). Survey respondents included social service organizations (53%), healthcare organizations (31%), and public health organizations (16%). The distribution of survey respondents was similar to the broader network (68% social service, 21% healthcare, and 10% public health). We present the implementation factors as they relate to overall themes.

**Table 1:** Network analysis survey results, *N* = 19.

Facilitators of engagement among partners^1^ (*n* = 19)	%	Common partnership activities^2^ (*n* = 10)	%	Successful aspects of MAMA’S referral process^3^ (*n* = 17)	%
Sharing resources among network members	60	Client referrals	90	Access to a point-person on the MAMA’S team	65
Network is responsive to needs of members	50	Case coordination and case conferencing	50	Coordinating and/or sharing patient information	59
Diverse and multidisciplinary network membership	40	Information exchange	50	Follow-up between MAMA’S staff and your agency	47
History of collaboration/sharing among network members	40	Advocacy/policy	40	Meetings to discuss service delivery/referral process	35
In-person meetings	40	Client assessments	40	Partnership with DHS hospitals and clinics	29
Regular meetings	40	Data sharing	40	Partnerships with other CBOs	29
Strong sense of trust among network members	20	Service delivery	40	Patient/client screening	29
Funding	0	Sharing resources	30	Patient care navigation with MAMA’S care coordinators	24
Technical assistance	0	Meetings/events/trainings	20		
Peer learning/sharing among networks partners	0	Technical Assistance	10		
Strong network leadership	0	Developing standards/procedures, tools or technologies, funding, and joint programming	0		

1 Respondents were asked to select the factors that improved their engagement in the MAMA’S Neighborhood Partnership.

2 Respondents were asked to indicate what their partnerships with other organizations entailed.

3 Respondents were asked to indicate which aspects of the MAMA’S Neighborhood referral process were working well.

Sample sizes varied because responses to these questions were optional.

### 3.1. Relationship-centered perinatal care

#### 3.1.1. MAMA’S neighborhood care coordinators embody a person-centered and place-based approach to perinatal care

The in-depth interviews revealed that MAMA’S Neighborhood staff, and specifically Care Coordinators, are central to the referral process, have a clear sense of the importance of addressing unmet social needs for improving maternal health outcomes, and view the care coordination process as an opportunity to mitigate social stressors to improve the overall circumstances of clients and their families:

We may think that we’re just linking people to resources but if you add that empowering too, I feel like that goes a much longer way than, ‘Hey, I’ll just connect you to a resource. You didn’t follow through with it. That’s on you.’ There’s more to it than that. It’s building that rapport with the patient to say, ‘Hey, you can trust me enough and maybe nobody else recognized all the hard work that you’re doing in your life, but I want to be the first to say, ‘Hey, I recognize you, I see you. I see the work that you’re doing. I see how hard that you’re fighting for your daughter or for your son or just to bring your family unit back together.’

Additionally, interview participants shared how the Care Coordinators’ extensive experience with and knowledge of community resources improve MAMA’S Neighborhood capacity to provide quality care. When asked what drives the selection of an organization for referral, this staff member describes how the quality of the service is their focus:

Yeah, it’s definitely the quality of the services. I like to get feedback from the patients. I also like to call these places on my own and kind of talk to somebody because I hate giving out resources that I don’t really know much about. I don’t want to be confused about what services exactly they offer because if I’m feeling confused, my patient is not going to know what’s going on. Just working the field for so long I’ve built some relationships with some of these places. So, I feel comfortable calling and saying, ‘Hey, I have somebody. Can I please send them?’ Or ‘Can we come together?’ So, it kind of works nicely that way. Yeah.

Similarly, 24% of network survey respondents recognized the role of MAMA’S Neighborhood staff in providing a person-centered approach to addressing unmet social needs (Table 1). One partner organization shared that the MAMA’S Neighborhood was most effective at improving their “cultural [and] holistic wrap-around services provision.”

#### 3.1.2. MAMA’S neighborhood care coordinator/client relationship as a resource for MAMA’S neighborhood partner organizations

MAMA’S Neighborhood staff described the relationships cultivated between Care Coordinators and clients as central to the referral process because Care Coordinators maintain “constant communication” with clients throughout the perinatal period. Forty-seven percent of respondents from MAMA’S Neighborhood partners shared that follow-up with MAMA’S staff (e.g., Care Coordinators) was a successful component of the referral process (Table 1). Further, as one survey respondent reflected: “MAMA’S [Neighborhood] has been successful in referring patients to programs as well as following up to find out status of that client’s referral. If additional information is needed or additional contact with client to obtain the information needed MAMA’S is good about assisting in communication.” However, a MAMA’S Neighborhood staff member shared that some partner organizations may not be proactively engaging MAMA’S staff enough:

Most of the time the partnering agencies are not reaching out to us, which really surprises me because we do spend a lot of time with our patients… at some point we’re seeing our patients every week. So, that is a huge disconnect that these agencies are not reaching out to MAMA’S for information or just for extra support. I find that I’m the one calling these places.

### 3.2. Barriers and facilitators of referrals through cross-sector partnerships

#### 3.2.1. Facilitator: designated point of contact for referrals

MAMA’S Neighborhood staff and partner organizations indicated that having a direct point of contact improved their ability to establish and complete referrals within the partnership. For instance, in the network survey, 65% of partner organizations reported that having access to a designated point person was a successful part of the referral process (Table 1). A staff member shared how having an established point of contact at a partner organization streamlined the referral completion process:

I would send it [referral] to the person that we’re designated to send it to, and I have never had a problem tracking a referral because they’ve been so helpful. I could just give her a call and “I can say oh, may I ask the status of this patient?” Or, I can just e-mail her and ask, “can I get the status of this patient? Is this patient receiving Nurse-Family Partnership?”

Additionally, some respondents described having onsite MAMA’S Neighborhood staff at their locations, staff responses to referral follow-up, and regular calls with staff to discuss client information and treatment planning improved communication throughout the referral process.

#### 3.2.2. Barrier: lack of shared referral tracking among MAMA’S neighborhood staff and partner organizations

Partner organizations described different approaches to internal referral tracking systems, such as tracking clients via phone calls and verbal check-ins or data-driven tracking platforms and varying degrees of integration with MAMA’S Neighborhood staff. For example, one partner organization reported that their counselors and case managers “work very closely with MAMA’S to meet all the patient needs in a collaboration of services.” While another described a sophisticated platform that was shared with MAMA’S staff:

Every referral that comes in gets entered into our database, and providers (like MAMA’S Neighborhood) get recorded. Our database allows us to run reports by provider which would give us the total amount of referrals received by MAMA’S Neighborhood. Each service is recorded into the database. Whether it is forms, notes, telephone calls, etc. The services received are documented either directly into the database or via forms which the nurse has documented the service/s received.

Alternatively, some partner organizations reported not having a structured process for tracking referrals among MAMA’S Neighborhood clients specifically: “We do not keep track of what agency the client is from unless it requires further assistance with the agency.” Further, partner organizations reported that 23% of partnerships entailed information sharing, while 18% entailed data sharing, suggesting that activity related to tracking between organizations is also limited (Table 1).

#### 3.2.3. Barrier: referral process tracking centered with MAMA’S care coordinators

Because Care Coordinators are broadly responsible for tracking referrals to partner organizations, they maintain much of the referral documentation, which may introduce communication challenges among MAMA’S staff. However, collaborative care meetings to address client needs among MAMA’S staff do ease some of these challenges. For instance, one clinician described how they “basically rely on either a social worker or the Care Coordinators to report back. Or at the collaborative care meeting to give an update on if the patient could connect with what we had discussed. Or, at the next time I see them for their appointment, we check in about that.” Care Coordinators often use informal verbal client check-ins to glean information on referral experiences or completion, which limits shared knowledge among MAMA’S Neighborhood staff about the quality of referral services: “I sometimes will just check in with the patient, see if they found it helpful.”

### 3.3. Strengths of a network approach to cross-sector partnership

#### 3.3.1. Bi-directional referrals among MAMA’S neighborhood partner organizations

For both Neighborhood staff and partner organizations, the network structure and partner collaboration facilitated a bi-directional referral process where MAMA’S Neighborhood staff are not the sole drivers of referrals to address unmet social needs within the partnership. For instance, 90% of partner organizations reported that client referrals are one of the most common activities among MAMA’S Neighborhood partner organizations, followed by case coordination and conferencing (50%, Table 1). Further, 29% of partner organizations indicated that their relationships with other organizations in the network were successful components of the MAMA’S Neighborhood referral process. Some partner organizations refer to each other when a referral is beyond their capacity to complete. As a MAMA’S Neighborhood staff member shared, “it’s not just the team within MAMA’S, but it’s even the collaborating agencies who we work with to help house our patients or to help put them in programs and things of that sort…We’re all working together as one.”

#### 3.3.2. Networking, training, and informational meetings

Among partner organization respondents, 35% agreed that meetings to discuss MAMA’S Neighborhood referral processes were a successful program component (Table 1). Additionally, 40% of respondents agreed that meetings among partner agencies were successful at facilitating their engagement in the Neighborhood partnership. MAMA’S Neighborhood staff shared several instances where attending training and informational meetings hosted by partner organizations facilitated their capacity to complete referrals by making more referral options available and creating greater transparency around referral steps for specific agencies and a direct point of contact:

We’ll get a Housing for Health training. And we are talking to and asking questions to the liaison, the person who we’re supposed to be in contact with when we want to refer a patient. That person is supposed to be able to address any issues we have, answer any questions, let us know if the applications that we’re putting in are complete, what other documentation we need so that application can go through successfully.

Opportunities to network and learn about new community resources also facilitated MAMA’S Neighborhood staff members’ abilities to expand the Neighborhood Partnership network:

We’re always looking for collaborative programs to help. We would email the different staff to say that there is going to be maybe a training or group session where the staff gets together and learns more about these different agencies. Anytime they would send an email, or it's put on our calendar, or we learn about it, we would go. That’s your time to network, to know about the different agencies or the different programs that are out there.

## 4. Discussion

This study aimed to examine the implementation of cross-sector partnerships for integrating social determinants in pregnancy care. Our analysis highlighted several implementation processes, facilitators, barriers, and outcomes that exemplify the success and opportunities of addressing unmet social needs through cross-sector partnerships. Our findings underscore the importance of cultivating and sustaining authentic human-centered relationships between patients, healthcare systems, and partner organizations.

Our study found that the relationships between MAMA’S Neighborhood Care Coordinators and clients facilitated the care coordination process by centering client needs and the relatability of MAMA’S Neighborhood Care Coordinators. Further, the longitudinal relationships that Care Coordinators established with clients throughout the perinatal period improved referral completion and communication among partner organizations, other staff, and clinicians in MAMA’S Neighborhood. These findings are similar to those of other studies on community health workers’ roles in improving patient navigation of social services systems, linkage to resources, and health outcomes (27–30). For instance, Kim et al. (27) found that community health worker interventions were effective among historically marginalized communities in improving access to preventive care. Additionally, Boyd and colleagues found that trust-based relationships between community health workers and perinatal women with chronic conditions improved engagement with the health system reduced stress, and improved health behaviors (28).

Beyond the process-related and intermediate improvements to referrals in cross-sector partnerships, the centrality of the Care Coordinator and client relationship in MAMA’S Neighborhood also speaks to the significance of relationship-based care as a mechanism to mitigate the impacts of structural racism, experiences of discrimination and implicit bias in the United States health care system and maternal health care (31–36). As Hardeman et al. (37) demonstrated, relationship-based clinical care provides opportunities for providers to be emotionally present, practice cultural humility, and facilitate reciprocal relationships with patients as experts on their health and well-being. For MAMA’S Neighborhood, many of these practices are inherent to Care Coordinators, given their positionality as members of clients’ communities and knowledge of community resources that align with clients’ needs.

The challenges related to shared tracking and siloed referral documentation among MAMA’S Neighborhood partner organizations and staff may reflect integration and partnership limits stemming from differences in organizational culture, resources, and capabilities (18, 38). Among MAMA’S Neighborhood staff, we found that documentation of referral outcomes and client experiences is informal, distinct from MAMA’s Neighborhood partner organizations, who use a variety of referral documentation processes. This ultimately presents challenges for shared outcome measurement (7). While opportunities to improve data sharing through digital platforms are essential for impact evaluation and process metrics (9), they may hinder aspects of relational communication that proved foundational to the MAMA’S Neighborhood program (e.g., having a direct point of contact at partner organizations, Care Coordinator relationships with clients). Thus, additional strategies may be needed to bridge gaps in shared tracking while upholding the autonomy and approaches to tracking that work best among partner organizations.

An important strength that emerged in MAMA’S Neighborhood is the network-based approach to integrating partner organizations alongside perinatal care through cross-sector partnerships. The network structure of the MAMA’S Neighborhood enables partner organizations to collaborate on referrals and for MAMA’S Neighborhood staff to network and learn about partner organizations’ referral processes, which increases transparency across organizations that might ordinarily be siloed. These types of relational structures in cross-sector partnerships are important for improving trust and learning within the partnership (9). Byhoff and Taylor (19) found that opportunities to facilitate shared learning and understanding within cross-sector partnerships are critical for continued buy-in among community-based organizations. Similar to other cross-sector partnerships, MAMA’S Neighborhood is initiated by a health system. However, the findings presented here show that the network approach also supports activities and referrals among partner organizations not created by the health system. Further, opportunities to engage with and learn from other partner agencies facilitate capacity building among partner organizations and MAMA’S Neighborhood staff, improving relationships’ depth and quality.

This study contributes to research on implementing cross-sector partnerships; however, it has important limitations. First, this study did not include data on the experiences and preferences of MAMA’S clients. This has important implications for defining the equitable, person-centered implementation of cross-sector partnerships in the MAMA’S Neighborhood network. For instance, it remains unclear whether participants shared the same views on MAMA’S relationship-centered approach or whether the quality of care received varied among partner organizations. We intend to elucidate client experiences in future research. Next, the study was conducted during the COVID-19 pandemic. Thus the themes that emerged may be related to the contextual impacts of the pandemic (e.g., limited in-person meetings or referrals within the partnership). While examining the impacts of COVID-19 was beyond the scope of this study, future research should describe how the pandemic shaped cross-sector partnerships, particularly as it relates to community resources and client needs. Second, this study did not consider variations in the relationships cultivated between MAMA’S Neighborhood staff and partner organizations, which may have depended on partner organizations’ characteristics (e.g., staff size, the scope of services, and proximity to services MAMA’S Neighborhood sites, etc.).

Lastly, the findings presented may be unique to California’s social and political environment, specifically Los Angeles County (a large region that covers diverse landscapes of available community resources), which may uniquely enable or hinder cross-sector partnerships, and thus results may not be generalizable to other settings. Despite these limitations, this study offers key evidence of the implementation successes and challenges of a robust cross-sector partnership serving historically marginalized communities during the perinatal period.

## Data availability statement

The data analyzed in this study is subject to the following licenses/restrictions: this data contain PHI. Requests to access these datasets should be directed to bridgette.blebu@lundquist.org.

## Ethics statement

The studies involving human participants were reviewed and approved by Los Angeles County Department of Public Health institutional review board (IRB # 2013-08-451). The participants provided their written informed consent to participate in this study.

## Author contributions

BB led the analysis and writing of this manuscript. PL supported the analysis and writing for this manuscript. WN, MH, AJ, and ES supported the writing and review for this manuscript and generated the original concepts for the overall project. All authors contributed to the article and approved the submitted version.

## Funding

This research was supported by the Robert Wood Johnson Foundation (RWJF), Agency for Healthcare Research and Quality (AHRQ), and Patient-Centered Outcomes Research Institute (PCORI; Grant Number K12 HS026407).

## Conflict of interest

The authors declare that the research was conducted without any commercial or financial relationships that could be construed as a potential conflict of interest.

## Publisher’s note

All claims expressed in this article are solely those of the authors and do not necessarily represent those of their affiliated organizations, or those of the publisher, the editors and the reviewers. Any product that may be evaluated in this article, or claim that may be made by its manufacturer, is not guaranteed or endorsed by the publisher.

## Author disclaimer

The content is solely the responsibility of the author(s) and does not necessarily represent the official views of RWJF, AHRQ, or PCORI.
