# Supplementary material for: Implementation of cross-sector partnerships: a description of implementation factors related to addressing social determinants to reduce racial disparities in adverse birth outcomes
Source: Front Public Health. 2023 Jun 16;11:1106740. doi: 10.3389/fpubh.2023.1106740 (PMC10313205; doi:10.3389/fpubh.2023.1106740)
Supplement: Supplementary file 1 [file Data_Sheet_1.docx]

| **Appendix 1.** List of MAMA'S Neighborhood Partner Organizations | |
| --- | --- |
| Partner Organization Name | Abbreviation |
| A New Vision For You | ANVFY |
| Angel Step Too, Volunteers of America | AST-VOA |
| Antelope Valley Birth Center | AVBC |
| Antelope Valley Department of Mental Health | AVDMH |
| Antelope Valley Green Thumb | AVGT |
| Antelope Valley Public Health | AVPH |
| Antelope Valley WIC | AVWIC |
| Black Infant Health | BIH |
| Catalyst Foundation | CF |
| Child & Family Center's Domestic Violence Program of the Antelope Valley | CFCDVP |
| Children’s Dental | CD |
| Children’s Law Center of California | CLCC |
| Christ Centered Ministries -Transitional housing facility | CCM1 |
| City of Inglewood Resources | CIR |
| Department of Children and Family Services (DCFS) | DCFS |
| Department of Mental Health | DMH |
| Didi Hirsch | DH |
| Doula Services, SBCC Community Based Doula Support | DS |
| East San Gabriel Valley Coalition for Homeless | ESGVHC |
| Fresh Start Transitional Home for Men | FSTHM |
| Grace Resources | GR |
| Harper Haven | HH |
| Holliday's Helping Hands | HHH |
| Homelight: Family Living | HFL |
| Housing For Health | HFH |
| Hygeia Breast Pumps | HBP |
| LA CADA Alice House | LAAH |
| LA County DMH Augustus Family Mental Health Center | LACAFMHC |
| LACDPH Monrovia Health Center | MHC |
| LAHSA Street Team - SPA 7 | LAHSAST |
| Legal Aid Foundation of Los Angeles | LAFLA |
| Los Angeles Job Corps | LAJC |
| Medical Legal Community Partnership | MLCP |
| Moms House | MH |
| Nurse Family Partnership | NFP |
| Options for Recovery | OR |
| Parents as Teachers | PT |
| Project 180 | P1801 |
| Project Joy, Inc. | PJ |
| Prototypes | PT1 |
| S.A.V.E.S | SAVES |
| SHIELDS for Families | SHIELDS |
| SPA 3 Whole Person Care Substance Use Disorder Program | SPA3 |
| Salvation Army | SA |
| St Joseph Church / St. Vincent DePaul Society | SJC |
| Street Medicine | SM |
| The Children Center of the Antelope Valley | TCCAV |
| Two-Lifestyles Women Empowerment Program | TLWEM |
| Valley Oasis | VO |
| Virtual MOMMY SUPPORT | VMS |
| Volunteers of America - Pomona | VOAP |
